# Higher dietary vitamin D intake is associated with better survival among older women: Results from the French EPIDOS cohort

**DOI:** 10.3389/fnut.2022.974909

**Published:** 2022-09-08

**Authors:** Jennifer Gautier, Jérémie Riou, Anne-Marie Schott, Hubert Blain, Yves Rolland, Patrick Saulnier, Cédric Annweiler

**Affiliations:** ^1^Department of Geriatric Medicine, Angers University Hospital, Angers University Memory Clinic, Research Center on Autonomy and Longevity, Angers, France; ^2^MINT, INSERM 1066, CNRS 6021, University of Angers, Bretagne Loire University, Angers, France; ^3^Delegation to Clinical Research and Innovation, Angers University Hospital, Angers, France; ^4^Department IMER, Lyon University Hospital, EA 4129, RECIF, University of Lyon, INSERM U831, Lyon, France; ^5^Department of Internal Medicine and Geriatrics, Montpellier University Hospital, University of Montpellier 1, Montpellier, France; ^6^Department of Geriatrics, Toulouse University Hospital, INSERM U1027, University of Toulouse III, Toulouse, France; ^7^UPRES EA 4638, University of Angers, UNAM, Angers, France; ^8^Department of Medical Biophysics, Schulich School of Medicine and Dentistry, Robarts Research Institute, The University of Western Ontario, London, ON, Canada

**Keywords:** vitamin D, mortality, eating, aged 75 and over, cohort studies

## Abstract

**Background:**

Hypovitaminosis D, a condition highly common among older adults, is associated with 35-percent increased all-cause mortality. In contrast, vitamin D supplementation prevents all-cause mortality. The possible role of the dietary intake of vitamin D on mortality remains yet unknown.

**Objectives:**

The objective of this prospective study was to determine all-cause mortality risk according to baseline dietary vitamin D intake among older adults while accounting for potential confounders including dietary calcium intake.

**Methods:**

Vitamin D and calcium dietary intakes were estimated at baseline from a self-administered food frequency questionnaire among 3,066 community-dwelling older women aged ≥75 years, recruited in the French EPIDOS cohort between 1992 and 1994, and for whom information about vital status was available in 2010. Dietary vitamin D and calcium intakes were defined as low if <400 IU/day or <1,200 mg/day, respectively.

**Results:**

The mean ± SD age of the whole cohort was 80.1 ± 3.6 years at baseline. The median survival time from baseline for participants with low dietary vitamin D intake was 11.5 years [95% confidence interval (CI): 11.0–11.9] vs. 12.2 years (95% CI: 11.7–12.9) for those consuming more than 400 IU/day (*p* = 0.003). Among those with calcium dietary intake <1,200 mg/day, a vitamin D consumption of 400 IU/day and over had a significant positive effect on all-cause mortality (RR: 0.86, *p* < 0.05). However, no association was retrieved between dietary vitamin D intake and all-cause mortality among participants with dietary calcium intake ≥1,200 mg/day.

**Conclusion:**

Higher dietary vitamin D intake was associated with better survival in the study cohort, specifically among those consuming <1,200 mg/day of dietary calcium.

## Introduction

Complementary alternative medicines (CAM), such as herbal products, dietary supplements, vitamins and minerals, are increasingly used by consumers to self-medicate in France, and all over the world. For instance, in Australia, the prevalence increased from 17.7 to 35.5% in only 3 years and the cod liver oil, containing vitamin D, was found to be the most popular (1). The use of this specific CAM or vitamin D supplements, which are readily available over the counter, represents a real support to fight against hypovitaminosis D in the general population.

In fact, hypovitaminosis D is a highly common condition (2), particularly in older adults (3, 4). Clinical interest is that vitamin D is a pluripotent steroid hormone with multiple bone and non-bone targets (2–5). In fact, vitamin D is involved in the health and function of multiple cells and organs, and hypovitaminosis D is accompanied by various diseases (2–5) and a greater risk of all-cause mortality in the short and long terms (6, 7). A prospective population-based study performed in the Netherland found that hypovitaminosis D was associated with all-cause mortality (HR: 1.88, 95% CI: 1.02–3.44) and cardiovascular mortality (HR: 5.33, 95% CI: 1.97–14.45) (8). In line, the correction of hypovitaminosis D with supplementation has shown beneficial effects on mortality (7, 9–12). For instance, a recent Cochrane review found that oral vitamin D supplementation reduced the risk of all-cause mortality in randomized placebo-controlled trials [relative risk (RR): 0.97, 95% CI: 0.94–0.99] (12). Specifically, the RR of cancer mortality was improved (RR: 0.88, 95% CI: 0.78–0.98). The regulation role of vitamin D in regulating immunity and its anti-inflammatory effects, are probably responsible for better survival (5). Nevertheless, this previous Cochrane review (12) found no association between vitamin D supplementation and cardiovascular mortality (RR: 0.98, 95% CI: 0.90–1.07) whereas a recent non-linear Mendelian randomization analysis reported a significantly higher risk in individuals with deficient vitamin D status (OR: 0.69, 95% CI: 0.52–0.92) (13).

The distinction in previous Cochrane review (12) was important since it highlighted that the relationship between vitamin D supplies and mortality could be more complex than initially expected. In fact, although vitamin D supplies may directly improve survival through its non-skeletal effects, it is noticeable that the increased intestinal absorption and tubular reabsorption of calcium attributable to vitamin D (2) has recently been associated with greater cardiovascular mortality (14, 15), possibly due to artery calcification (16). In line, Mendelian randomization analyses have shown that genetically predicted lifelong higher concentrations of serum calcium may shorten life expectancy and increase cardiovascular disease risk (17). This may explain the lack of benefits of vitamin D on cardiovascular mortality in previous Cochrane review (12), and prompts to account for calcium intake while examining the impact of vitamin D on survival.

Another important issue is that vitamin D supplements are not the only source of vitamin D (2, 3), and that only 14% of the elderly population is supplemented with vitamin D (18). Thus, the effect of the dietary intake seems decisive. Surprisingly, and to the best of our knowledge, the effects of the dietary vitamin D intake on all-cause mortality has not yet been examined. We hypothesized that a greater dietary vitamin D intake could predict better survival in older adults without excessive intake of calcium. Our objective was to determine the risk of all-cause mortality according to baseline dietary vitamin D intake among community-dwelling women aged 75 and older, while taking into account all potential confounders including the dietary calcium intake.

## Materials and methods

### Study overview

This study used part of the data collected for the French EPIDOS (EPIDémiologie de l'OStéoporose) survey in Amiens, Lyon, Montpellier, Paris and Toulouse since 1992. The survey included 7,598 women living in the community aged 75 and over, who were recruited between February 1992 and April 1994. First, these women were randomly selected from voter lists and several health insurance registries, and then, asked to volunteer for the survey. Participants unable to walk independently and those who had history of hip fractures or bilateral hip replacements were excluded. The details of the sampling and data collection procedures can be found elsewhere (19). Finally, the present analysis considered data on 3,066 women when: (*a*) ≥75 years old, (*b*) community-dwelling, (*c*) assessment of dietary intakes at baseline, (*d*) full data on mortality up to 12 October 2010 (excluding data from Paris and Amiens), and (*e*) full data regarding EPIDOS variables.

All participants were included after giving written informed consent for the research. The study was performed in accordance with the ethical principles outlined by the Helsinki Declaration. The local Ethics Committee of each city approved the entire study protocol.

### Dietary intake of vitamin D

The weekly dietary vitamin D intake was estimated at baseline of the EPIDOS study from a self-administered food frequency questionnaire, modified from Fardellone's (20), as previously published (21–23). This food frequency questionnaire with 21 questions, included 2 seafood items [lean fish (fresh, canning or frozen), fat fish (herring, anchovy, salmon, sardine, cod liver, mackerel)], 6 dairy items (milk, cream and yogurt desserts, as well as cream, baked and soft cheeses), and some other questions about products that were also used in the calculation of vitamin D intake (eggs, fruits and vegetables, starchy foods, chocolate, drinking and meat) For each participant, the mean dietary intake of vitamin D, calculated in μg/week, was obtained by multiplying the content of each food by the frequency of consumption and summing it all. The CIQUAL Database, which is regularly updated by the French food safety agency (AFSSA), was used to determine the vitamin D content of each food items (24). In the present study, we used the threshold value of 400 IU/day (i.e., 10 μg/day or 70 μg/week) to define low and adequate (i.e., high enough) dietary vitamin D intake, according to the recommended dietary intake (RDI) of vitamin D for the French adult population (25).

### Mortality

These were collected either by telephone or through a search of the French national death registry CépiDC (Centre d'épidémiologie sur les causes médicales de décès) up to 12 October 2010. Women still alive on 12 October 2010 were right-censored.

### Covariates

The following covariates were included in the data analysis: center of recruitment, calcium dietary intake, protein dietary intake, body mass index (BMI), cognitive disorders, sun exposure at midday, functional autonomy, hypertension, diabetes mellitus, angina pectoris, cancer, smoking, polypharmacy, regular use of vitamin D supplements and the regular use of calcium supplements.

The calcium and protein intakes were estimated from the food frequency questionnaire using the same approach as for the vitamin D assessment. Dietary calcium intake was categorized into 3 groups: <900; 900–1,200; ≥1,200 mg/day according to the French RDI (25). An intake of 900 mg/day is the minimum calcium intake recommended for adults, and the cut-off of 1,200 mg/day is the minimum intake recommended for older adults. Protein (in g/kg/day) was considered as a continuous variable as it respected the log-linearity hypothesis. Anthropometric measures (i.e., height and weight) were acquired to calculate the BMI (measure of weight in kilograms divided by height in meters squared). Global cognitive function was based on the Pfeiffer's Short Portable Mental State Questionnaire (SPMSQ; score 0–10, best) (26), a reliable standardized validated screening test for organic brain syndromes. Cognitive disorders were defined as a SPMSQ score below 8 (27). Sun exposure at midday (yes/no) was asked, corresponding to a minimum 15-min exposure to sunlight between 11_AM_ and 3_PM_ with face and hands uncovered. Functional autonomy was measured using the Instrumental Activities of Daily Living scale (IADL; score 0–8, best) and considered normal for a score of at least 7 (28). Evaluation of chronic diseases and smoking was based on self-report. Information about hypertension, diabetes mellitus, angina pectoris and cancer were especially obtained from this standardized question: “Do you currently suffer from hypertension, diabetes mellitus, angina pectoris or cancer?”. Finally, women were asked to bring all the medication they were regularly taking to the clinical center. Polypharmacy was defined as using 5 drugs or more per day. The regular use of vitamin D and calcium supplements during the previous year was also sought (yes/no).

### Statistical analysis

Firstly, a descriptive analysis of the participants' characteristics was performed using effectives and percentages for qualitative variables, and means ± standard deviations or medians [inter-quartile range (IQR)], as appropriate, for quantitative variables.

Comparisons were performed according to the dietary vitamin D intake lower or higher than 400 IU/day using chi^2^ or Fisher exact test, as appropriate, for qualitative variables, and Student's *t-*test or Mann Whitney Wilcoxon test according to the normal distribution assumption for quantitative variables. We used a Cox proportional hazards regression models stratified on the recruitment center to determine the association of the dietary vitamin D intake with all-cause mortality, using the level <400 IU/day as the reference group. Univariate analysis was firstly performed to determine variables associated with the mortality. They were selected for the final model if the *p*-value for association was below 20%. The search for interactions was performed from the final model. Only interactions clinically relevant and interpretable were examined. The log-linearity assumption has been checked for each continuous variable and a conversion into qualitative variable was performed if this assumption was not verified. Analyses were performed using SAS®, version 9.4 (SAS Institute Inc.). All tests were 2-sided, and *p* < 0.05 was considered statistically significant. For multiple comparisons, when it is necessary *p*-values were adjusted using Hochberg procedure, which allows a control of the Family Wise Error Rate (FWER) at 0.05.

## Results

**Figure 1:**
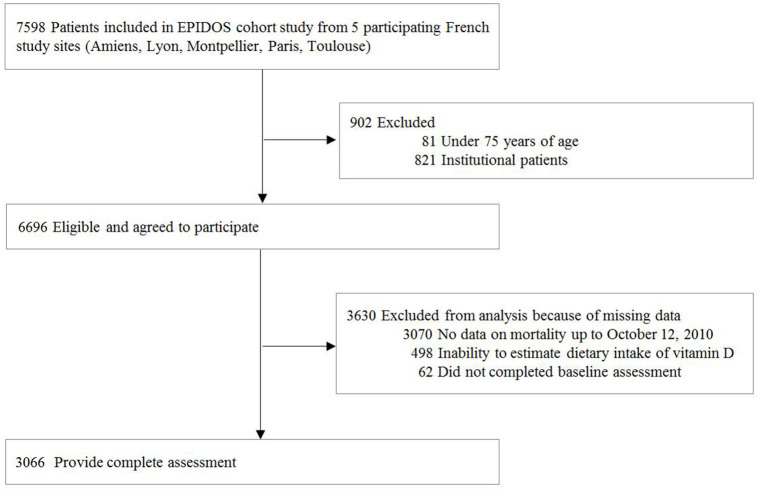
Participants flowchart.

Of 7,598 EPIDOS participants at 5 study sites, 3,066 women had all data available and were included in the present analysis (Figure 1).

The mean age was 80.1 ± 3.6 years (mean ± SD), the mean dietary intake of vitamin D was estimated at 350 ± 170 IU/day, and the mean duration of the follow-up was 11.3 ± 5.2 years. Of the 3,066 women, 1,966 (64%) had low dietary vitamin D intake <400 IU/day. Table 1 describes the participants' characteristics and the comparison of the two groups of interest defined by the dietary vitamin D intake estimate (<400 IU/day vs. ≥400 IU/day). Compared to women with vitamin D intake ≥ 400 IU/day, those with low vitamin D intake were significantly older (*p* < 0.001) and had lower dietary calcium intake (*p* < 0.001) and lower dietary protein intake (*p* < 0.001). In addition, more women died before 12 October 2010 in the group with the low vitamin D intake (80.5 vs. 76.7%, *p* = 0.02).

**Table 1:** Characteristics of study participants (*n* = 3,066).

	**Total** **(*n* = 3,066)**	**Dietary intake of vitamin D**		*p-*value*
		** <400 IU/day (*n* = 1,966)**	**≥400 IU/day (*n* = 1,100)**	
Recruitment center, *n* (%)				<0.001
Lyon	1,044 (34)	646 (33)	398 (36)	
Montpellier	1,005 (33)	601 (31)	404 (37)	
Toulouse	1,017 (33)	719 (37)	298 (27)	
Age at baseline, years	80.1 ± 3.6	80.3 ± 3.6	79.7 ± 3.5	<0.001
Vitamin D dietary intake, IU/day	350 ± 170	250 ± 90	540 ± 140	-
Duration of the follow-up, years	11.3 ± 5.2	11.1 ± 5.2	11.7 ± 5.2	0.004
Death on 12 October 2010, *n* (%)	2,426 (79.1)	1,582 (80.5)	844 (76.7)	0.02
Calcium dietary intake, mg/day	950 ± 340	880 ± 310	1,060 ± 370	<0.001
Protein dietary intake, g/kg/day	1.2 ± 0.4	1.2 ± 0.3	1.3 ± 0.4	<0.001
BMI, kg/m^2^	25.5 ± 4.0	25.5 ± 4.0	25.4 ± 4.2	0.55
Cognitive disorders^†^, *n* (%)	315 (10)	212 (11)	103 (9)	0.21
Sun exposure at midday^‡^, *n* (%)	1,599 (52)	1,023 (52)	576 (52)	0.86
Functional autonomy^¶^, *n* (%)	2,276 (74)	1,450 (74)	826 (75)	0.42
Hypertension, *n* (%)	1,389 (45)	894 (45)	495 (45)	0.80
Diabetes mellitus, *n* (%)	174 (5.7)	116 (5.9)	58 (5.3)	0.47
Angina pectoris, *n* (%)	536 (17)	341 (17)	195 (18)	0.79
Cancer, *n* (%)	152 (5.0)	104 (5.3)	48 (4.4)	0.26
Smoking, *n* (%)				0.17
Former	289 (9.4)	171 (8.7)	118 (10.7)	
Current	95 (3.1)	63 (3.2)	32 (2.9)	
Polypharmacy||, *n* (%)	1,735 (57)	1,089 (55)	646 (59)	0.07
Use vitamin D supplements, *n* (%)	469 (15)	298 (15)	170 (15)	0.86
Use calcium supplements, *n* (%)	614 (20)	365 (19)	249 (23)	0.007

Data are n (%) or mean ± SD; IU, international unit; BMI, body mass index (measure of weight in kilograms divided by height in meters squared); IADL, instrumental activities of daily living scale. ^*^Chi^2^ or Fisher exact test for qualitative variables, Student's t-test or Wilcoxon-Mann-Whitney test for quantitative variables. ^†^Pfeiffer Short Portable Mental State Questionnaire score below 8 out of 10. ^‡^Regular exposure of more than 15 min to the sunlight (face and hands uncovered) between 11_AM_ and 3_PM_ when the weather is nice. ^¶^Instrumental Activities of Daily Living score of 7 or more out of 8. ||Use of 5 drugs or more per day.

**Figure 2:**
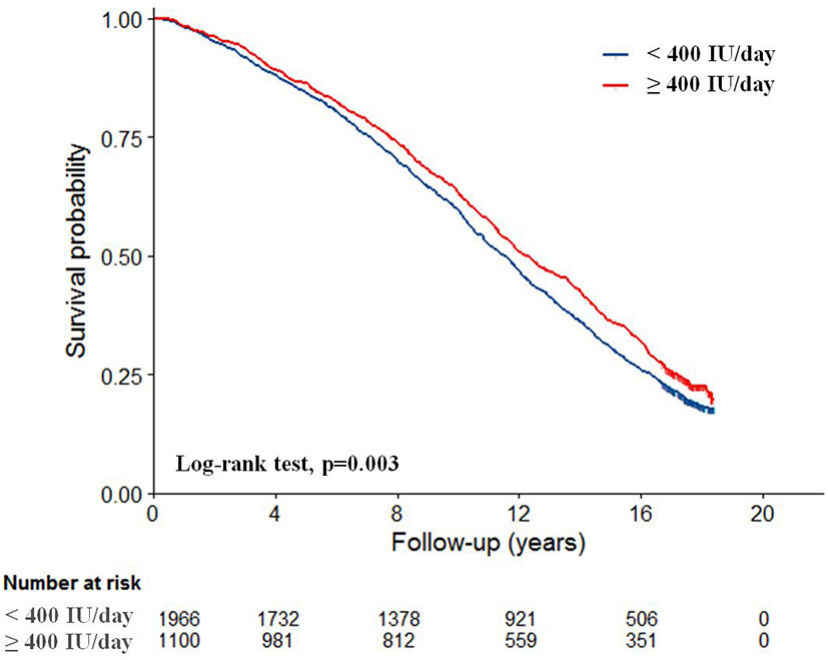
Kaplan-Meier survival curves according to dietary vitamin D intake (*n* = 3,066).

The median survival time from baseline for women with low dietary vitamin D intake was 11.5 years (95% CI: 11.0–11.9) vs. 12.2 years (95% CI: 11.7–12.9) for those consuming more than 400 IU/day (*p* = 0.003). Estimates of survival function for all participants by dietary vitamin D group are shown in Figure 2.

Table 2 describes the results of the Cox proportional hazard regression model stratified on the recruitment center. We found that, for women with dietary calcium intake <900 mg/day, those consuming ≥400 IU/day of vitamin D exhibited a reduced risk of all-cause mortality (RR: 0.86, 95% CI: 0.75–0.98, *p* = 0.02) after adjustment for the potential confounders. Similarly, among the women consuming between 900 and 1,200 mg of calcium per day, we obtained similar results, those having ≥400 IU/day of vitamin D exhibiting a reduced risk of all-cause mortality (RR: 0.86, 95% CI: 0.74–0.999, *p* = 0.046). Nevertheless, among the women with dietary calcium intake ≥1,200 mg/day, there was no association of dietary vitamin D intake with all-cause mortality (*p* = 0.18). Finally, we also found an increased mortality risk for participants with a higher dietary protein intake (RR: 1.15, 95% CI: 1.01–1.32, *p* = 0.04), with cognitive disorders at baseline (RR: 1.38, 95% CI: 1.21–1.57, *p* < 0.001), with hypertension (RR: 1.14, 95% CI: 1.05–1.24, *p* = 0.003), with angina pectoris (RR: 1.33, 95% CI: 1.20–1.48, *p* < 0.001), with diabetes mellitus (RR: 1.45, 95% CI: 1.23–1.71, *p* < 0.001), for smokers (RR: 1.38, 95% CI: 1.10–1.73 for current smoker and RR: 1.17, 95% CI: 1.02–1.34 for former smoker, compared to non-smoker at baseline, *p* = 0.003) and also with polypharmacy (RR: 1.13, 95% CI: 1.13–1.24, *p* = 0.006). Finally, dependent women with IADL score <7 but who exposed themselves to the sun at midday had a decreased mortality risk (RR: 0.75, 95% CI: 0.63–0.88, *p* < 0.001); an association not retrieved among independent women with IADL score of 7 and more.

**Table 2:** Association of dietary vitamin D intake with all-cause mortality (*n* = 3,066).

	**Cox model stratified on recruitment center**	
	**RR (95% CI)**	***p*** **-Value**
Vitamin D dietary intake ≥400 IU/day		
with calcium dietary intake <900 mg/day	0.86 (0.75–0.98)	0.02
with calcium dietary intake [900–1,200 mg/day]	0.86 (0.74–0.999)	0.046
with calcium dietary intake ≥1,200 mg/day	1.13 (0.94–1.36)	0.18
Protein dietary intake (per 1 g/kg/day)	1.15 (1.01–1.32)	0.04
Cognitive disorder*	1.38 (1.21–1.57)	<0.001
Sun exposure at midday^†^		
with IADL score ≥ 7/8	0.93 (0.84–1.02)	0.13
with IADL score <7/8	0.75 (0.63–0.88)	<0.001
Hypertension	1.14 (1.05–1.24)	0.003
Diabetes mellitus	1.45 (1.23–1.71)	<0.001
Angina pectoris	1.33 (1.20–1.48)	<0.001
Cancer	1.14 (0.95–1.36)	0.16
Smoking		0.003
Former	1.17 (1.02–1.34)	
Current	1.38 (1.10–1.73)	
Polypharmacy^‡^	1.13 (1.04–1.24)	0.006
Use vitamin D supplements	0.94 (0.81–1.08)	0.34
Use calcium supplements	1.02 (0.90–1.16)	0.77

IU, international unit; CI, confidence interval; IADL, instrumental activities of daily living scale; RR, relative risk. ^*^Pfeiffer Short Portable Mental State Questionnaire score below 8 out of 10. ^†^Regular exposure of more than 15 min to the sunlight (face and hands uncovered) between 11_AM_ and 3_PM_ when the weather is nice. ^‡^Use of 5 drugs or more per day.

## Discussion

The main finding of this population-based cohort study is that, irrespective of all measured potential confounders, the dietary vitamin D intake was inversely associated with all-cause mortality among this large studied sample of French older women followed during 17 years on average; an association found only for those with low-to-moderate, but not high, dietary calcium intake. These findings may provide insight into the benefits of vitamin D intake on health and longevity in older adults, which could be more complex than originally expected and depend on vitamin-calcium metabolism.

Highlighting benefits of vitamin D on mortality risk is consistent with prior literature. In particular, previous prospective observational studies assessed the association of circulating 25-hydroxyvitamin D (25(OH)D) concentration with life expectancy. The results consistently showed that the lower the 25(OH)D concentration, the greater the all-cause mortality risk (29, 30). Bolland et al. found in a meta-analysis of 32 studies that the HR for all-cause mortality comparing the lowest (0–9 ng/mL) to the highest (>30 ng/mL) category of 25(OH)D was 1.9 (*p* < 0.001) (29). Similarly, Gaksch et al. found in an Individual Participant Data meta-analysis including 26,916 participants and using standardized measurements of 25(OH)D, that the HR for all-cause mortality comparing the lowest (0–12 ng/mL) to the highest (≥30 ng/mL) category of 25(OH)D was 1.8 (95% CI: 1.4–1.9) (30). These results were strengthened by those of randomized controlled trials (RCTs) that tested the effect of vitamin D supplementation on mortality. Overall, the meta-analyses of RCTs suggested that vitamin D supplementation may moderately, yet statistically significantly, reduce mortality (7, 9–12). Thus, the findings of the current study provide additional information by showing that not only supplementation, but also dietary intake, was associated to better survival, with a clinically relevant magnitude of effect. The results of this study are fully consistent with the recommendations of Pilz and al. who promote food fortification to improve vitamin D status in the general population, as it has already been introduced in the United States, Canada, India and Finland (31). What is more, and since the primary role of vitamin D is to promote intestinal absorption of calcium, the study had the merit to underline for the first time to our knowledge that the effects of vitamin D intake on mortality depend on concomitant calcium intake: although effective in the case of low-to-moderate calcium intake, the beneficial effects of vitamin D intake disappeared with higher calcium intake. The latter finding is consistent with recent literature showing that increased calcium intake leads to an increased risk of incident cardiovascular events and mortality (14, 15). For instance, a high dietary calcium intake >1,400 mg/day was associated with higher all-cause mortality (HR: 1.4, 95% CI: 1.2–1.7) among more than 60,000 postmenopausal women from the Swedish Mammography Cohort (15). Similarly, some RCTs have reported an increased risk of cardiovascular events from calcium supplementation, and a subsequent meta-analysis has confirmed that supplementation was responsible for increased event rates (29, 32). Also, our present results confirm that vitamin D intake should not be considered without taking into account the micronutrients, like calcium, whose metabolism is linked to vitamin D and could attenuate the magnitude of its beneficial effects.

How vitamin D is associated to mortality is not fully elucidated. On the one hand, our results could be explained by reverse causation i.e., that the dietary vitamin D intake was reduced due to underlying diseases, or by confounding. Potential confounding factors such as inflammation or loss of autonomy are associated with both poor nutrition and increased mortality (33). In the present analysis, the association between low dietary vitamin D intake and mortality remained, however, significant despite careful statistical adjustments. Alternatively, the association could be causal because vitamin D is a secosteroid hormone that exhibits multiple biological actions mediated by the vitamin D receptor present in almost all human tissues (2–5). Since vitamin D is needed for the regulation of cellular growth, differentiation and function (2–5), lower vitamin D status leads to multiple organ dysfunction, disability and unstable health status, which are all causes of deconditioning and mortality. Moreover, vitamin D has antitumor properties including inhibition of malignant cell proliferation, angiogenesis or metastasis (34), as well as inhibition of cancer-related inflammation (35). It is also shown that vitamin D, as well as having regulating immunity role, anti-inflammatory effects and anti-endotoxin properties, activates immune cells to produce antimicrobial peptides such as cathelicidins and defensins, which reduces viability of viruses and bacteria (36, 37). Recent literature also showed that daily vitamin D supplementation to maintain serum 25(OH)D>100 nmol/L was associated with a reduced risk of diabetes in prediabetic adults (38), and with an improved control of blood pressure in hypertensive individuals (39). Of note, it should be acknowledged that, since most of the effects of vitamin D involve calcium metabolism, this may explain why, in the present study, the beneficial effects of vitamin D intake disappeared while it was accompanied by excessive absorption of calcium. Our results suggest that it is preferable (a) to privilege a correct intake of vitamin D in older adults to exert skeletal and non-skeletal effects of vitamin D, and (b) to accompany vitamin D intake with real but careful calcium intake to ensure phosphocalcic metabolism without generating non-skeletal adverse effects; a strategy already used by rheumatologists in the field of osteoporosis (40).

Besides the association between dietary vitamin D intake and mortality, the current study also showed that the chronic conditions such as smoking, hypertension, diabetes mellitus, angina pectoris, cognitive disorders and polypharmacy were associated with a greater mortality risk during the follow-up. These findings are consensual and consistent with previous literature showing a decrease in life expectancy in these people compared to healthy older adults (41–43). Thus, they validate the consistency of our results and reinforce the credibility of our main result of an inverse association between the dietary intake of vitamin D and all-cause mortality.

The strengths of the present study include (a) the originality of the research question on common dietary habits, (b) the standardized collection of data, (c) the long-term follow-up of a large sample of community-dwelling older women in three different large French areas, and (d) the detailed description of the participants' characteristics allowing the analysis of potential interactions as well as the use of multiple models to measure adjusted associations. Regardless, a number of limitations also existed. First, the cohort might be unrepresentative of the general population of older adults due to the restricted inclusion of well-functioning older women who may have easy access to vitamin D-rich foods. For instance, 74% were independent, and the mean BMI was higher than 25, which indicates overweight according to the National Heart, Lung, and Blood Institute (44). Moreover, the study participants may have been more motivated, with a greater interest in personal health issues, than the general population of older adults. Secondly, although we were able to control for important characteristics that could modify the associations, residual potential confounders, including the variables changes during follow-up or the plasma parathyroid hormone level, might still be present. Thirdly, the use of an observational design prevents any causal inference from the present study. Fourthly, the difficulty in summarizing dietary habits -especially with regards to the dietary vitamin D intake- explains the failure to consider this association in the past. An additional limitation therefore lied in the dietary assessment method, which was initially not built to determine the dietary intake of vitamin D (20). Fifthly, self-reported data could be problematic when studying cognitively impaired populations due to misreporting difficulty.

## Conclusion

We found a decreased mortality risk among older women with dietary intake of vitamin D above 400 IU/day *and* dietary intake of calcium below 1,200 mg/day. This encourages the use of vitamin D-rich foods or foods fortified with vitamin D, and calls for precautions toward calcium intake. Further prospective observational cohorts and randomized clinical trials are needed to confirm the impact of vitamin D-calcium intakes on all-cause and cause-specific mortality in a variety of adult populations and care settings.

## Data availability statement

The raw data supporting the conclusions of this article will be made available by the authors, without undue reservation.

## Ethics statement

The studies involving human participants were reviewed and approved by Local Ethical Committees of Amiens, Lyon, Montpellier, Paris and Toulouse (France). The patients/participants provided their written informed consent to participate in this study.

## Investigators of EPIDOS study

Coordinators: Breart, Dargent-Molina, Meunier, Schott, Hans, and Delmas. Principal investigators: Baudoin and Sebert (Amiens); Chapuy and Schott (Lyon); Favier and Marcelli (Montpellier); Hausherr, Menkes and Cormier (Paris); Grandjean and Ribot (Toulouse).

## Author contributions

CA has full access to all of the data in the study, takes responsibility for the data, the analyses and interpretation, the conduct of the research, has the right to publish any and all data, separate and apart from the attitudes of the sponsor, and had a supervision role. CA and JG contributed to the study concept and design. A-MS obtained funding, contributed to the acquisition of data, provided administrative, technical, or material support. JG, JR, PS, and CA analyzed and interpreted the data. JG, CA, and JR drafted the manuscript, which was critically revised for important intellectual content by A-MS, HB, YR, and PS. All authors contributed to the article and approved the submitted version.

## Funding

This work was supported by the French Ministry of Health. The sponsor had no role in the design and conduct of the study, in the collection, management, analysis, interpretation of the data or in the preparation, review, or approval of the manuscript.

## Conflict of interest

The authors declare that the research was conducted in the absence of any commercial or financial relationships that could be construed as a potential conflict of interest.

## Publisher's note

All claims expressed in this article are solely those of the authors and do not necessarily represent those of their affiliated organizations, or those of the publisher, the editors and the reviewers. Any product that may be evaluated in this article, or claim that may be made by its manufacturer, is not guaranteed or endorsed by the publisher.
